# Editorial: Isolation, structural elucidation, and biological evaluation of bioactive products from traditional medicine

**DOI:** 10.3389/fchem.2022.984399

**Published:** 2022-08-11

**Authors:** Qi-Meng Zhu, Juan Zhang, Cheng-Peng Sun

**Affiliations:** ^1^ College of Pharmacy, Dalian Medical University, Dalian, China; ^2^ School of Pharmaceutical Sciences, Health Science Center, Shenzhen University, Shenzhen, China

**Keywords:** natural products, biological activity, structure-activity relationship, structural elucidation, traditional medicine

The culture of traditional Chinese medicines (TCMs) is extensive and profound in China, and the TCMs application traces back to thousands of years. As a treasure, TCMs play an immeasurable role in the treatment of various diseases, such as Parkinson’s disease, kidney disease, and acute lung injury. During the last decade, thousands of natural products with novel structures and unique bioactivities have been isolated from traditional medicines, including kurarinone with an inhibitory effect against the soluble epoxide hydrolase activity, (23*S*)-11*β*,23-dihydroxy-8*α*,9*β*,14*β*-dammar-13 (17)-ene-3,24-dione with an agonistic effect toward the farnesoid X receptor, and alismanin A with an agonistic effect toward the pregnane X receptor. The vigorous development of life sciences promotes the development of new methods and the discovery of novel targets that are used to evaluate the biological functions and potential molecular targets of natural products from TCMs. Meanwhile, the discovery of new structures sharing unprecedented skeletons riches the structural diversity of natural products, as well as elucidates the new mechanisms of action (MOAs), providing a new avenue for the drug discovery. Therefore, the team of Frontiers in Chemistry initiates the Research Topic based on the “*Isolation, Structural Elucidation, and Biological Evaluation of Bioactive Products from Traditional Medicine*.” This Research Topic in Frontiers in Chemistry compiles 20 articles in this Research Topic.

Firstly, an article from Ming et al. demonstrated the discovery of four new guaiane-type sesquiterpenoids and two known analogues from leaves of *Artemisia argyi* Lévl et Vant, and found that some sesquiterpenoids possessed potent antiproliferative activities for A549, MCF-7, and HepG2 cells.

Secondly, Yan et al. found nine new sesquiterpenes, including eurylosesquiterpenosides A–D and eurylosesquiterpenols E–I, in the investigation on the ultraviolet b (UVB) irradiation protective constituents of *Oplopanax elatus*. They applied UVB induced HaCaT cells to explore the anti-photoaging mechanism, and demonstrated that these compounds inhibited the expression of matrix metalloproteinase-1 (MMP-1), increased the collagen I expression, and reduced the p38 phosphorylation level and release of tumor necrosis factor-α and cyclooxygenase-2, which revealed the potential MOA involved in reducing MMP-1 expression and down-regulating the production of inflammatory cytokines in UVB-induced HaCaT cells.

Han et al. reported the presence of eleven new lupanes, elesesterpenes A–K, from leaves of *Eleutherococcus sessiliflorus*, and identified the structures of all the isolated compounds by spectroscopic data and X-ray diffraction. Some of them were found to exhibit remarkable anti-inflammatory and anti-proliferative activities.

A study by Xu et al. reported eight new alkaloids isolated from *Stemona tuberosa* together with 21 known compounds. The structures of all new compounds were determined through multiple spectroscopic means, including pyridine solvent effect, X-ray single-crystal diffraction, and Mosher method. The isolated compounds showcased anti-inflammatory effects in LPS-induced RAW264.7 cells.

Wang et al.’s group reported a pair of 3,4-seco-cycloartane triterpenoid isomers sharing a rare peroxy bridge, xuetonins A and B, from *Kadsura heteroclita* together with six new and forty-three known analogues. The subsequent investigation demonstrated their inhibitory effects against rheumatoid arthritis fibroblast-like synoviocytes (RAFLS) and hepatoprotective effects.

Finally, Fei et al. discovered an unprecedented tetracyclic diterpenoid with functional groups of a 6/6/5-fused tricyclic ring and a 4,5-dimethyldihydrofuran-2(3*H*)-one, caesalpinbondin A, form seeds of *Caesalpinia bonduc,* and proposed its possible biogenetic pathway*.* Based on the assay in the model of Alzheimer’s disease (AD) of *Caenorhabditis elegans,* caesalpinbondin A showed an anti-AD potential.

The editors hope that the articles compiled in this publication will be worthwhile for the researchers working on the field of bioactive natural products from traditional medicines, and that they will contribute to understand the biological activities and applications of natural products. For this reason, we would like to thank the effort carried out by all those who contribute to this Research Topic: authors, co-authors, reviewers, and the team of Frontiers in Chemistry.

